# Safety and Efficacy of LockeT Suture Retention Device for Large‐Bore Venous Access Closure

**DOI:** 10.1111/jce.70303

**Published:** 2026-04-03

**Authors:** Pedro J. Diaz Delgado, Aashish Katapadi, Eli Herink, Karnik Patel, Donita Atkins, Rajesh Kabra, Naga Venkata K. Pothineni, Douglas Darden, Rakesh Gopinathannair, Dhanunjaya Lakkireddy

**Affiliations:** ^1^ Kansas City Heart Rhythm Institute Overland Park Kansas USA; ^2^ HCA Midwest Overland Park Kansas USA

**Keywords:** ambulation, electrophysiology, hemostasis, large bore, left atrial appendage occlusion, lockeT

## Abstract

**Background:**

Electrophysiology (EP) procedures increasingly require large‐bore venous access (LBVA), which is associated with a higher risk of vascular complications. While vascular closure devices (VCDs) are well established for arterial access, data on venous closure—particularly for LBVA—remain limited. The LockeT device is a suture‐mediated venous closure system designed to achieve rapid hemostasis without intravascular components, but its performance in LBVA has not been well characterized.

**Methods:**

We conducted a single‐center, retrospective observational study of patients undergoing EP procedures requiring LBVA (> 14 French) between June 2023 and October 2024. Patients undergoing left atrial appendage occlusion (LAAO) or leadless pacemaker (LPM) implantation with LockeT venous closure were included. Primary endpoints were effectiveness, assessed by hemostasis at 2 h (HA2H) and time to hemostasis (TTH), and safety, assessed by major and minor vascular complications. Secondary outcomes included time to ambulation (TTA) and same‐day discharge (SDD).

**Results:**

A total of 139 patients were included (median age 76.0 years; 55.4% male). LockeT deployment was successful in 97.0% of cases. HA2H was achieved in 100% of successfully deployed cases. Median TTH was 12 s (IQR: 0.0–78.0), and median TTA was 4.1 h (IQR: 3.0–5.1). Same‐day discharge occurred in 91.4% of patients. No major vascular complications were observed. Minor complications occurred in 3.6% of patients and included oozing and mild hematoma. Device failure occurred in 2.8% of cases, all during early adoption, and was managed successfully with manual compression. Outcomes did not differ by procedure type or baseline oral anticoagulation status.

**Conclusions:**

In this single‐center experience, LockeT demonstrated high procedural success, rapid hemostasis, early ambulation, and low complication rates for venous closure following EP procedures requiring LBVA. These findings support LockeT as a feasible and effective venous closure strategy in contemporary EP practice, though prospective studies with comparator groups and longer follow‐up are warranted.

**Abbreviations**
EP: electrophysiology
Fr: French
HA2H: hemostasis at two hours
LAAO: left atrial appendage occlusion
LBVA: large bore venous access
MC: manual compression
OAC: oral anticoagulation
TTA: time to ambulation
TTD: time to discharge
TTH: time to hemostasis
VCD(s): vascular closure devices(s)

## 1 Introduction

**Figure 1:**
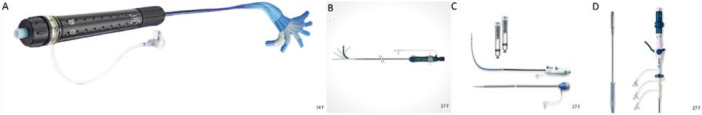
Large bore vascular access sheaths. Multiple sheaths range from 14 to 27 Fr, increasing risk of access‐related complications. (A) Abbott Amulet steerable sheath, (B) Boston Scientific Watchman steerable sheath, (C) Medtronic Micra sheath/delivery system and (D) Abbott Aveir sheath/delivery system.

Catheter‐based electrophysiologic (EP) procedures are central to some of the most effective therapies for arrhythmias and require vascular access, for which postprocedural hemostasis is critical. Yet, this exposes patients to the risk of vascular complications such as hematomas, pseudoaneurysms, arteriovenous fistulas, and blood loss [1]. These risks are closely associated with the sheath size utilized, specifically large‐bore vascular access (LBVA) > 12 Fr, which increases the likelihood of complications (Figure 1) [2].

Manual compression (MC) has been the traditional method for achieving hemostasis [3]. While MC is effective, it is resource‐intensive, requiring healthcare personnel to apply physical pressure, and it requires prolonged bed rest, which can be uncomfortable and frustrating for patients. In response to these challenges, various vascular closure devices (VCDs) with both active and passive mechanisms have been developed to enhance postprocedural management. Most VCDs have been developed for arteriotomy closure rather than venous access. However, several venous closure devices, including Vascade MVP and Perclose, have been evaluated and have demonstrated promising results for venous closure.

Perclose ProGlide (Abbott Vascular Devices) has been studied in procedures requiring large‐bore venous access (LBVA), including transcatheter mitral valve edge‐to‐edge repair and transcatheter mitral valve replacement using 16–24 Fr sheaths. These studies demonstrated favorable efficacy and safety outcomes for venous vascular access closure [4].

In another study comparing Perclose ProGlide with figure‐of‐eight sutures for LBVA (> 13 Fr sheaths), device‐based closure was associated with shorter time to hemostasis and earlier ambulation [5].

**Figure 2:**
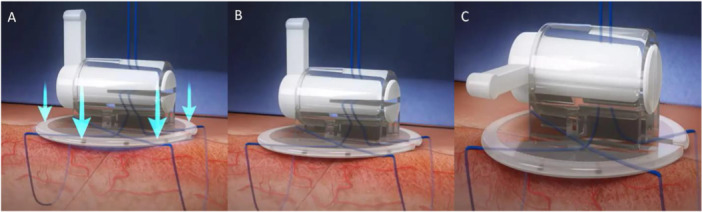
LockeT device. The LockeT device is applied to the skin over the access site (A), and the suture is threaded through the stopcock (B). The stopcock is then rotated clockwise (C) to apply tension.

Some VCD‐based approaches employ a mechanism conceptually similar to the stopcock technique used with the figure‐of‐eight suture, which is the LockeT (Catheter Precision, Fort Mills, SC, USA; Figure 2)—which has demonstrated adequate hemostasis along with minimal complications [6, 7].

Most studies involving VCDs have focused on < 12 Fr sheaths, but several EP procedures now require larger sheath sizes [8, 9, 10]. Although few VCDs exist for LBVA, their effectiveness is still relatively less well published in comparison to arterial devices [10]. While the safety and effectiveness of LockeT is studied in smaller‐sized vascular access management, its effectiveness for LBVA has not been evaluated in depth. Consequently, this study aims to evaluate the performance of the LockeT device in EP procedures involving LBVA.

## 2 Methods

### 2.1 Study Design

We performed a single‐center (Overland Park Regional Medical Center, Overland Park, KS, USA), a retrospective observational study involving patients who underwent EP procedures requiring LBVA, defined as greater than 14 Fr sheaths. This included all patients who underwent left atrial appendage occlusion (LAAO) or leadless pacemaker (LPM) implantation with LockeT use for postprocedural venous closure. The study was approved by the Institutional Review Board and adhered to the ethical principles outlined in the Declaration of Helsinki.

All procedures were performed using ultrasound‐guided venous access. Periprocedural anticoagulation management differed by procedure type. In patients undergoing LAAO, oral anticoagulation (OAC) was continued uninterrupted, and intravenous heparin was administered during the procedure to achieve a target activated clotting time (ACT) > 350 s. Upon completion of the procedure, protamine sulfate (100 units) was administered for heparin reversal, with additional dosing as needed to achieve an ACT < 200 s. In contrast, patients undergoing LPM implantation typically were not receiving chronic OAC; however, when OAC was present, it was continued uninterrupted. LPM patients received a single intraprocedural dose of 5000 units of intravenous heparin without routine ACT monitoring or protamine reversal.

### 2.2 Patient Selection

The study included all patients who underwent planned outpatient LAAO, urgent inpatient LAAO, or LPM implantation between June 2023 and October 2024 with LockeT used for venous access closure. Patients were excluded if they met any of the following criteria: (1) age < 18 years, (2) arterial access on the ipsilateral side of LockeT use, (3) body mass index > 50 kg/m^2^, (4) sheath size < 12 Fr, (5) intraprocedural complication unrelated to vascular closure, (6) pre‐existing hematoma, aneurysm, local or systemic infection, or (7) known hypercoagulable states, including prothrombotic disorders, thrombocytopenia (platelet count < 150 000), or other bleeding disorders.

### 2.3 Outcomes and Definitions

The primary objectives were to evaluate the safety profile and effectiveness of LockeT for venous closure in LBVA procedures. Effectiveness was assessed by hemostasis at two hours (HA2H) and time to hemostasis (TTH). HA2H was defined as the absence of bleeding or oozing—defined as a very slow, nonpulsatile, superficial bleeding that blots the gauze over a period of time but does not require intervention—at 2 h postprocedure. TTH was defined as the time from removal of the final sheath to LockeT deployment with immediate cessation of bleeding. Confirmation of HA2H was ensured via standardized nursing documentation and postprocedural vascular assessments.

Safety was evaluated by the incidence of major and minor complications. Major complications included bleeding resulting in a > 2 g/dL decrease in hemoglobin, hemodynamic compromise, transfusion requirement, moderate‐to‐large hematoma, acute limb ischemia, fistula or aneurysm formation, or significant groin pain requiring opioid administration. Minor complications included oozing or mild hematoma formation not requiring intervention.

Secondary outcomes included same‐day discharge (SDD) rate and time to ambulation (TTA), defined as the time from removal of the final sheath to the first documented independent or assisted ambulation. Patients were monitored throughout their hospital course only; no short‐term or long‐term postdischarge follow‐up was performed.

### 2.4 LockeT Venous Closure

**Figure 3:**
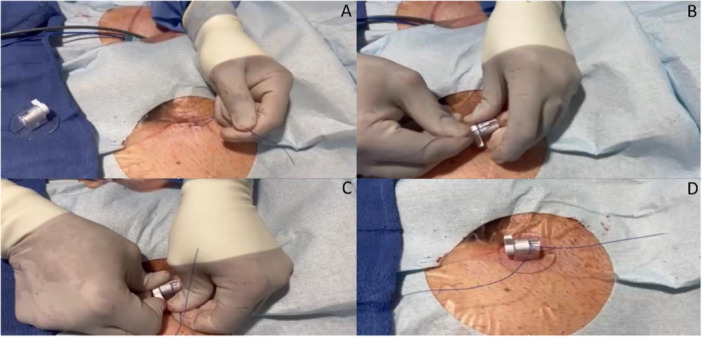
Application of LockeT. The LockeT device step by step application (A) Create a standard figure of eight and remove sheaths. Clean site via standard protocol. (B) Tighten and place sutures in LockeT. (C) Apply pressure and rotate the handle 90. (D) Observe hemostasis. Follow regular protocol.

The LockeT utilizes a modified purse‐string suture technique, as previously described [4]. Briefly, following removal of the final sheath, a suture is applied and fed through the LockeT device. As tension is applied, the suture ends are guided through the LockeT stopcock while the base is positioned over the access site. The stopcock is rotated counterclockwise to achieve controlled tightening without knot tying (Figure 3 and Video 1). The translucent base permits direct visualization of the access site during deployment. One LockeT device was used per groin site regardless of the number of venous access points or sheaths. Local anesthetic (e.g., lidocaine or bupivacaine) was administered at the access site per operator preference.

**Video 1:** This video demonstrates step by step application of the modified figure‐8 suture, deployment of the Locket device and tightening the suture to collapse the tissue track created by the large bore venous sheath, this creates hemostasis and allows for the clotting mechanism to kick in and create durable hemostasis without any foreign material placed into the vessel wall.

### 2.5 Statistical Analysis

Continuous variables are reported as mean ± standard deviation or median with interquartile range (IQR), as appropriate. Categorical variables are reported as counts and percentages. Analysis of variance or Kruskal–Wallis testing was used for continuous variables, and Pearson's *χ* ^2^ test was used for categorical variables. A *p* < 0.05 was considered statistically significant. All analyses were performed using SPSS version 30.0.0 (IBM, Armonk, IL, USA).

## 3 Results

### 3.1 Baseline and Procedural Characteristics

A total of 139 patients were included, with a median age of 76.0 years (IQR: 62.0–82.0); 55.4% (*n* = 77) were male. The median body mass index was 28.2 kg/m^2^ (IQR: 24.8–32.5). Common comorbidities included hypertension (68.3%, *n* = 95), coronary artery disease (23.7%, *n* = 33), and diabetes mellitus (22.3%, *n* = 31). Procedures included LAAO in 82.7% (*n* = 115) and LPM implantation in 17.3% (*n* = 24). Sheath sizes used were 14 Fr (82.7%, *n* = 115), 18 Fr (7.9%, *n* = 11), and 23 Fr (9.4%, *n* = 23). Ultrasound‐guided venous access was performed in all cases.

Baseline anticoagulation data were available for the majority of patients undergoing LAAO (85 of 117 patients). Among these patients, 70 (82.4%) were receiving OAC at baseline, including 65 (92.9%) on a direct oral anticoagulant and 5 (7.1%) on warfarin. Baseline anticoagulation data were not consistently available for patients undergoing LPM implantation and therefore could not be reliably analyzed for this subgroup. Additional baseline characteristics are summarized in Table 1.

**Table 1:** Baseline and procedural characteristics.

	% (*N*) or median (IQR)
Age (years)	76.0 (IQR: 62.0–80.0)
Male gender	55.4% (77)
Body Mass Index (kg/m^2^)	28.2 (IQR: 24.8–32.5)
Race	
White	88.5% (123)
Black	2.9% (4)
Hispanic	1.4% (2)
Asian	2.2% (3)
Other	5.0% (7)
Hypertension	68.3% (95)
Diabetes	22.3% (31)
Coronary artery disease	23.7% (33)
Congestive heart failure	5.8% (8)
Chronic kidney disease	6.5% (9)
Type of atrial fibrillation	
Paroxysmal	67.6% (94)
Early persistent	7.9% (11)
Longstanding persistent	5.8% (8)
Type of procedure	
LAAO	82.7% (115)
Leadless PPM	17.3% (24)
Maximum sheath size	
14 Fr	82.7% (115)
18 Fr	7.9% (11)
23 Fr	9.4% (13)

Abbreviations: Fr, French; IQR, interquartile range; Kg/m^2^, kilograms per squared meter; LAAO, left atrial appendage occlusion; PPM, permanent pacemaker.

### 3.2 Hemostasis and Efficacy Outcomes

LockeT deployment was successful in 97.0% (135/139) of cases. HA2H was achieved in 100% (*n* = 135) of successfully deployed cases. Median TTH was 12 s (IQR: 0.0–78.0). Median TTA was 4.1 h (IQR 3.0–5.1), and SDD was achieved in 91.4% (*n* = 127). Time to discharge was not recorded as a continuous variable. The eight patients who were not discharged on the same day were admitted for observation due to late procedural timing rather than vascular access‐related issues.

No differences in TTH, HA2H, or complication rates were observed between LAAO and LPM procedures, nor between patients on versus off OAC. Intracardiac echocardiography was used on a case‐by‐case basis, occasionally requiring bilateral venous access; no differences in closure dynamics, TTH, or in‐hospital complication rates were observed between unilateral and bilateral approaches (Table 2).

**Table 2:** Efficacy and safety outcomes.

Variables	% (*N*) or median (IQR)
Hemostasis at 2 h	97.1% (135)
Time to hemostasis (min)	0.2 (IQR: 0.0–1.3)
Time to ambulation (h)	4.1 (IQR: 3.0–5.1)
Same day discharge	91.4% (127)
Access site complications	3.6% (5)
Device failure	2.9% (4)

Abbreviations: h, hours; IQR, interquartile range; Min, minutes.

### 3.3 Major and Minor Complications

No major vascular complications occurred. Minor complications occurred in 3.6% of patients and included three cases of oozing and two cases of mild hematoma formation. There were four device failures (2.8%) due to suture breakage or improper deployment, all of which occurred during the early adoption phase of LockeT use at our institution and were attributed to excessive suture tension and operator inexperience. All device failures were successfully managed with manual compression without impact on SDD or other in‐hospital outcomes.

## 4 Discussion

In this retrospective, single‐center study, we evaluated the role of the LockeT device for LBVA closure following LPM and LAAO procedures. Our principal findings demonstrate that LockeT was associated with low complication rates, rapid TTH, early ambulation, and a high rate of same‐day discharge SDD. These findings support LockeT as a feasible and effective venous closure strategy in contemporary electrophysiologic procedures requiring LBVA.

The LockeT device employs a suture‐mediated mechanism conceptually similar to the stopcock technique used with figure‐of‐eight sutures, which has previously been described for venous LBVA closure [6]. However, LockeT incorporates several design features that extend beyond ease of use. The rounded retention plate allows for a more uniform distribution of compressive force across the access site, while the adjustable suture mechanism permits controlled titration of compression and the ability to retighten the suture if hemostasis is incomplete prior to device removal. This design also minimizes direct skin contact and may reduce skin abrasion commonly associated with traditional stopcock‐based figure‐of‐eight sutures. In addition, LockeT allows direct visualization of the access site and can be used with multiple sheaths regardless of the number of ipsilateral groin access points, which may simplify postprocedural management and improve workflow efficiency.

Several venous VCDs, including Perclose ProGlide (Abbott Cardiovascular, Chicago, IL) and Vascade MVP (Haemonetics, Boston, MA), have been evaluated in procedures involving LBVA, most commonly with sheath sizes ranging from 12 to 14 Fr. Prior studies have demonstrated that these devices can reduce TTH and TTA compared with figure‐of‐eight sutures, with generally acceptable safety profiles [4, 5, 11, 12, 13], For example, venous closure with Vascade in LAAO procedures has been associated with significantly shorter TTH compared with figure‐of‐eight sutures, and Perclose ProGlide has demonstrated reductions in both TTH and TTA in LBVA ≥ 13 French [5, 13]. Among studies reporting outcomes of venous closure strategies, Perclose ProGlide has been associated with complication rates of approximately 20%, whereas figure‐of‐eight sutures have been associated with complication rates of up to 35% [14]. In contrast, cumulative complication rates with LockeT have previously been reported at 6.5% [7]. In our cohort, LockeT similarly demonstrated high success in achieving hemostasis, with a TTH of 0.2 min, facilitating early mobilization and SDD.

The reported mean TTA of 4.1 h in our study should be interpreted in the context of early institutional experience with the device. Although hemostasis was typically achieved shortly after sheath removal, patients were maintained on an additional conservative period of bed rest prior to ambulation, resulting in the observed TTA. Notably, sutures were released approximately 2 h before ambulation. In comparison, the SAFE‐VEIN trial—the only other study specifically evaluating venous VCDs in LBVA greater than 13 Fr—reported a mean TTA of approximately 5.6 h with Perclose ProGlide, although individual sheath sizes were not specified. These findings suggest that LockeT compares favorably with existing venous closure strategies in LBVA, even within a conservative early adoption framework.

No major vascular complications occurred in association with LockeT use in this cohort. Device failure due to suture breakage occurred in a small proportion of cases and represented the only significant complication observed. All failures occurred during the early adoption phase and were attributed to excessive suture tension during deployment, reflecting a learning curve with appropriate tensioning. All cases were managed successfully with manual compression, without impact on SDD or other in‐hospital clinical outcomes. The observed device failure rate was consistent with previously reported venous VCD failure rates, which range widely depending on device type [7, 15].

Unlike some venous closure devices that rely on collagen‐based or thrombin‐containing bioabsorbable materials, LockeT achieves hemostasis by compressing the subcutaneous tissue tract without directly altering venous wall integrity. This mechanism may be particularly advantageous in venous access, where preservation of vessel patency and minimization of local tissue reaction are desirable, especially in patients who may require repeat access.

Cost considerations are increasingly relevant as the volume of LBVA procedures continues to rise. Direct cost comparisons across venous VCDs are inherently limited by institutional variability in contractual agreements, purchasing structures, and vendor negotiations. Despite this heterogeneity, LockeT was consistently associated with lower acquisition costs at our institution compared with other commercially available venous VCDs, while supporting high SDD rates and reduced resource utilization. Although the magnitude of cost differences may not be generalizable across institutions, these findings suggest that LockeT represents a practical and cost‐efficient option for venous LBVA closure.

### 4.1 Study Limitations

This study has several limitations. It was a single‐center, retrospective observational analysis conducted in a relatively small cohort, which may limit generalizability. Follow‐up was restricted to the index hospitalization, with no short‐ or long‐term postdischarge follow‐up performed. Time to discharge was not captured as a continuous variable; instead, SDD rate was used as a clinically relevant surrogate. Standardized pain assessments, analgesic or narcotic utilization, and sheath inner versus outer diameter measurements were not systematically collected and therefore could not be analyzed.

LockeT deployment may be operator‐ and experience‐dependent, as most closures were performed by operators with substantial procedural experience, introducing potential selection bias and influence from institutional practice patterns. The absence of a comparator group limits assessment of relative efficacy, and procedures involving larger sheath sizes, such as those used for PFA, were not evaluated. Despite these limitations, this study represents the first evaluation of LockeT for LBVA and provides preliminary safety data. Prospective studies with comparator groups, broader sheath size inclusion, longer follow‐up, and incorporation of patient reported outcomes are warranted.

## 5 Conclusion

LBVA presents a challenge for vascular closure, with an increased risk of complications. In our study of procedures requiring LBVA, LockeT provided effective hemostasis with minimal complications. However, more studies are still needed to evaluate its effectiveness in various EP procedures requiring LBVA. There is a lack of a comparator group in our study, which limits the interpretability of the efficacy of the LockeT. However, other studies that are currently underway have the presence of a comparator group, where this aspect will be evaluated.

## Funding

The authors received no specific funding for this work.

## Disclosure

Dr. Kabra is a consultant for Abbott, Alta Thera, and Biosense Webster. Dr. Pothineni is a consultant for Medtronic and Biosense Webster. Dr. Gopinathannair is a consultant for Boston Scientific and Abbott. Dr. Lakkireddy is a consultant for Abbott, Atricure, Biosense Webster, Boston Scientific, LifeTech Sciences, and Medtronic. All other authors have no disclosures.

## References

[1] M. Avvedimento , C. Real , J. Nuche , et al., “Incidence, Predictors, and Prognostic Impact of Bleeding Events After TAVR According to VARC‐3 Criteria,” JACC: Cardiovascular Interventions 16 (2023): 2262–2274. 37676226 10.1016/j.jcin.2023.07.005

[2] D. E. Haines , S. Beheiry , J. G. Akar , et al., “Heart Rhythm Society Expert Consensus Statement on Electrophysiology Laboratory Standards: Process, Protocols, Equipment, Personnel, and Safety,” Heart Rhythm: Official Journal of the Heart Rhythm Society 11 (2014): e9–e51. 10.1016/j.hrthm.2014.03.042 PMC7106221 24814989

[3] S. Mohanty , C. Trivedi , S. Beheiry , et al., “Venous Access‐Site Closure With Vascular Closure Device vs. Manual Compression in Patients Undergoing Catheter Ablation or Left Atrial Appendage Occlusion Under Uninterrupted Anticoagulation: A Multicentre Experience on Efficacy and Complications,” EP Europace 21 (2019): 1048–1054. 10.1093/europace/euz004 30726903

[4] M. Mohammed , P. Nona , E. Abou Asala , et al., “Preclosure of Large Bore Venous Access Sites in Patients Undergoing Transcatheter Mitral Replacement and Repair,” Catheterization and Cardiovascular Interventions: Official Journal of the Society for Cardiac Angiography & Interventions 100, no. 1 (July 2022): 163–168, 10.1002/ccd.30229. 35568977

[5] M. Ali , F. Masood , L. Erickson , et al., “Suture Closure AFtEr Large Bore Vein Access (SAFE‐VEIN): A Randomized, Prospective Study of the Efficacy and Safety of Venous Closure Device,” Catheterization and Cardiovascular Interventions 104, no. 4 (October 2024): 820–828, 10.1002/ccd.31173. 39087741

[6] J. Payne , S. Aznaurov , and S. Gautam , “Three‐Way Stopcock Suture Technique for Hemostasis After Ablation for Atrial Fibrillation,” Journal of Cardiovascular Electrophysiology 29, no. 12 (December 2018): 1724–1727, 10.1111/jce.13712. 30106208

[7] A. Katapadi , N. Pham , N. Chelikam , et al., “Feasibility, Safety, and Efficacy of a Novel External Compression Vascular Closure Device: The LockeT Study,” Journal of Cardiovascular Electrophysiology 1952, no. 35 (May 2024): 1952–1959. 10.1111/jce.16381 39099135

[8] I. Ben‐Dor , P. Craig , R. Torguson , et al., “MynxGrip Vascular Closure Device Versus Manual Compression for Hemostasis of Percutaneous Transfemoral Venous Access Closure: Results From a Prospective Multicenter Randomized Study,” Cardiovascular Revascularization Medicine 19 (2018): 418–422. 29656937 10.1016/j.carrev.2018.03.007

[9] A. Natale , S. Mohanty , P. Y. Liu , et al., “Venous Vascular Closure System Versus Manual Compression Following Multiple Access Electrophysiology Procedures,” JACC: Clinical Electrophysiology 6 (2020): 111–124. 31971899 10.1016/j.jacep.2019.08.013

[10] J. A. Shaw , E. Dewire , A. Nugent , and A. C. Eisenhauer , “Use of Suture‐Mediated Vascular Closure Devices for the Management of Femoral Vein Access After Transcatheter Procedures,” Catheterization and Cardiovascular Interventions 63 (2004): 439–443. 15558775 10.1002/ccd.20190

[11] A. M. Ierardi , A. Coppola , M. Renzulli , et al., “Effectiveness and Safety of Different Vascular Closure Devices: Multicentre Prospective Observational Study,” Cardiovascular and Interventional Radiology 46 (2023): 827–834. 37225968 10.1007/s00270-023-03463-5 PMC10208551

[12] L. Paraggio , F. Bianchini , C. Aurigemma , et al., “Femoral Large Bore Sheath Management: How to Prevent Vascular Complications From Vessel Puncture to Sheath Removal,” Circulation. Cardiovascular interventions 17 (2024): e014156. 39166330 10.1161/CIRCINTERVENTIONS.124.014156 PMC11404769

[13] H. Golzarian , K. T. Graebel , R. Bailey , et al., “Efficacy and Safety of the VASCADE MVP Venous Vascular Closure Device in Patients Undergoing Percutaneous Left Atrial Appendage Occlusion With Watchman,” Catheterization and Cardiovascular Interventions 104 (2024): 1260–1266. 39279138 10.1002/ccd.31209

[14] H. Lodhi , S. Shaukat , A. Mathews , B. Maini , and H. Khalili , “Comparison of Figure‐of‐Eight Suture and Perclose ProGlide Suture‐Mediated Closure in Large Bore Venous Access Hemostasis: A Randomized Controlled Trial,” American Journal of Cardiology 209 (2023): 181–183. 37863115 10.1016/j.amjcard.2023.09.105

[15] S. Bangalore , N. Arora , and F. S. Resnic , “Vascular Closure Device Failure: Frequency and Implications: A Propensity‐Matched Analysis,” Circulation: Cardiovascular Interventions 2 (2009): 549–556. 20031773 10.1161/CIRCINTERVENTIONS.109.877407 PMC3046770

